# Vaccine Safety and Efficacy Evaluation of a Recombinant Bovine Respiratory Syncytial Virus (BRSV) with Deletion of the SH Gene and Subunit Vaccines Based On Recombinant Human RSV Proteins: N-nanorings, P and M2-1, in Calves with Maternal Antibodies

**DOI:** 10.1371/journal.pone.0100392

**Published:** 2014-06-19

**Authors:** Krister Blodörn, Sara Hägglund, Jenna Fix, Catherine Dubuquoy, Boby Makabi-Panzu, Michelle Thom, Per Karlsson, Jean-Louis Roque, Erika Karlstam, John Pringle, Jean-François Eléouët, Sabine Riffault, Geraldine Taylor, Jean François Valarcher

**Affiliations:** 1 Swedish University of Agricultural Sciences, Host Pathogen Interaction Group, Department of Clinical Sciences, Uppsala, Sweden; 2 INRA, Unité de Virologie et Immunologie Moléculaires, Jouy-en-Josas, France; 3 The Pirbright Institute, Pirbright, Surrey, United Kingdom; 4 National Veterinary Institute, Department of Virology, Immunology, and Parasitology, Uppsala, Sweden; 5 Clinique Veterinaire des Mazets, Riom es Montagnes, France; 6 National Veterinary Institute, Department of Pathology and Wildlife Diseases, Uppsala, Sweden; Imperial College London, United Kingdom

## Abstract

The development of safe and effective vaccines against both bovine and human respiratory syncytial viruses (BRSV, HRSV) to be used in the presence of RSV-specific maternally-derived antibodies (MDA) remains a high priority in human and veterinary medicine. Herein, we present safety and efficacy results from a virulent BRSV challenge of calves with MDA, which were immunized with one of three vaccine candidates that allow serological differentiation of infected from vaccinated animals (DIVA): an SH gene-deleted recombinant BRSV (ΔSHrBRSV), and two subunit (SU) formulations based on HRSV-P, -M2-1, and -N recombinant proteins displaying BRSV-F and -G epitopes, adjuvanted by either oil emulsion (Montanide ISA71^VG^, SUMont) or immunostimulating complex matrices (AbISCO-300, SUAbis). Whereas all control animals developed severe respiratory disease and shed high levels of virus following BRSV challenge, ΔSHrBRSV-immunized calves demonstrated almost complete clinical and virological protection five weeks after a single intranasal vaccination. Although mucosal vaccination with ΔSHrBRSV failed to induce a detectable immunological response, there was a rapid and strong anamnestic mucosal BRSV-specific IgA, virus neutralizing antibody and local T cell response following challenge with virulent BRSV. Calves immunized twice intramuscularly, three weeks apart with SUMont were also well protected two weeks after boost. The protection was not as pronounced as that in ΔSHrBRSV-immunized animals, but superior to those immunized twice subcutaneously three weeks apart with SUAbis. Antibody responses induced by the subunit vaccines were non-neutralizing and not directed against BRSV F or G proteins. When formulated as SUMont but not as SUAbis, the HRSV N, P and M2-1 proteins induced strong systemic cross-protective cell-mediated immune responses detectable already after priming. ΔSHrBRSV and SUMont are two promising DIVA-compatible vaccines, apparently inducing protection by different immune responses that were influenced by vaccine-composition, immunization route and regimen.

## Introduction

Bovine respiratory syncytial virus (BRSV), a pneumovirus in the family *Paramyxoviridae*, is a major cause of respiratory disease in young calves [1]. By causing high morbidity and mortality, this virus impacts dramatically on animal welfare and productivity, resulting in significant economic losses to farmers, and is even a public health-concern through the risk of antibiotic-resistance developing from the massive use of antibiotics to treat secondary bacterial infections [2].

Clinical signs of respiratory disease are modulated by the presence of BRSV-specific immunity and since BRSV seroprevalence is high in adult cattle [3], disease is mainly observed in young calves, even in the presence of low to moderate levels of BRSV-specific maternal antibodies (MDA) [4]. Although high levels of MDA protect against disease, even low levels impact negatively on the development of humoral immune responses induced by BRSV vaccination or infection in calves [5]. This, and the inherent immaturity of the immune system of young animals, is a hurdle that needs to be overcome in developing a protective vaccine for this target group [6].

Similar to BRSV in cattle, human (H)RSV, is a major cause of acute lower respiratory infection in humans, which in turn is a leading cause of child morbidity and mortality worldwide [7]. Although host-specific, BRSV and HRSV are genetically and antigenically closely related, and have a similar pathogenesis and clinical expression upon infection of calves and young children, respectively [1]. The similarity of these viruses makes research into either field complementary, and is utilized in vaccine development.

The RSV genomes consist of a single stranded, negative-sense RNA containing ten genes encoding eleven proteins [1]. Among these proteins, the fusion protein F (F) and the glycoprotein G (G) induce protective humoral responses [8], [9], in the form of virus-neutralizing systemic antibodies [10], and mucosal IgA [11]. Mucosal IgA can be induced in the respiratory tract by parenteral immunization [12], but mucosal immunization is more likely to prime mucosal immunity in the presence of MDA [13]. Furthermore, the F, N, M2 [14] and P (G. Taylor, unpublished observations) proteins are recognized by bovine CD8+ T cells, known to be important for clearance of BRSV and clinical recovery [15].

Knowledge of these protein-specific immunogenic characteristics is essential to develop effective vaccines to control RSV. Although extensive research has been performed to develop RSV vaccines, no vaccine is yet commercially available for humans [16], largely due to the potential for vaccine-induced exacerbation of HRSV disease by inactivated vaccines, first reported by Kim et al. in 1969 [17]. Conversely, several BRSV vaccines have been commercially available since the 1970’s. The early modified live BRSV vaccines were attenuated by serial cell culture passage, and were exclusively for parenteral use until 2007 when one became licensed for intranasal (i.n.) use due to its greater efficacy in calves with MDA [18]. However, intranasal administration of a modified live vaccine carries the risk of spread and reversion to virulence, and the use of modified live vaccine has been associated with exacerbated clinical BRSV disease when administered in presence of a natural BRSV infection [19].

For this reason, and to achieve greater protective durability, as well as cost-efficiency and practicality of vaccine handling, adjuvanted inactivated vaccines have also been developed and commercialized. Although these inactivated BRSV vaccines have been used very extensively, there is evidence of exacerbated BRSV disease upon natural infection of vaccinated calves, similar to that in man, which has also been experimentally reproduced [20], [21]. Despite the concurrent development of adjuvants that can induce balanced immune responses, which may avoid disease exacerbation and increase efficacy, none of these commercially available, classic inactivated BRSV vaccines have proven to be completely effective against severe experimental challenge when administrated to young calves in the presence of BRSV-specific MDA [22].

To improve both BRSV and HRSV vaccine efficacy and safety, genetic engineering has been used to produce DNA vaccines, vectored vaccines and attenuated candidate strains with mutations and/or deletions in one of six viral genes (NS1, NS2, SH, G, F and M2-1) [1], [23]. One of the most promising HRSV vaccine candidates is an attenuated strain containing a combination of three temperature sensitive (*ts*) phenotypical point-mutations, in addition to deletion of the SH gene, which have shown sufficient attenuation and indications of protection in clinical trials in infants [23]. Its bovine counterpart, a recombinant BRSV with a deletion of the SH gene, is attenuated in calves and has been shown to be safe and to induce good protection in colostrum-restricted calves [24]. However, the use of genetically modified viruses is not always acceptable, even if the modification is made by deletion, which drastically reduces the risk of reversion to wild-type. Therefore, new inactivated vaccines are also required, such as subunit vaccines containing RSV-specific proteins and epitopes [12], [25]. Both these vaccine approaches have the added advantage that by omitting virus genes or proteins, vaccinated animals can be serologically distinguished among infected (DIVA). Thus, vaccination does not interfere with seroepidemiological surveillance. Furthermore, due to the natural and gradual genetic changes in circulating BRSV, the DIVA characteristic is essential to track vaccine safety and efficacy in the field at a population level.

In the current study, we have evaluated and compared the safety, immunogenicity and protective potential of an attenuated recombinant BRSV lacking the SH gene (ΔSHrBRSV) using i.n. administration, and a subunit vaccine (SU) composed of recombinant RSV proteins. The SU, which consisted of HRSV-M2-1 and -P proteins, and nanorings with 10 or 11 protomers of HRSV-N protein displaying BRSV-F and -G epitopes, was evaluated following formulation with two different adjuvants, and was administered either intramuscularly (i.m.) or subcutaneously (s.c.). Protection afforded by immunization was determined in a virulent BRSV challenge, five weeks after first vaccination.

## Materials and Methods

### Ethics Statement

The experiment was carried out in compliance with the E.U. Directive 86/609, and approved by the Ethical Committee of the district court of Uppsala, Sweden (Ref. no. C330/11).

### Cells and Viruses

Bovine turbinate (BT) cells, fetal calf kidney (CK) cells and Vero cells were propagated at low passages, as previously described [12], [26]. All cells were free from bovine viral diarrhea virus (BVDV) and mycoplasmas.

Deleted SH recombinant BRSV (ΔSHrBRSV) was derived from full-length cDNA of BRSV strain A51908 [27], variant Atue51908 (GenBank accession no. AF092942), as previously described [28]. Stocks of ΔSHrBRSV were prepared in Vero cell monolayers and verified to be free of BVDV, as previously described [29].

Virulent BRSV used for the experimental challenge was prepared using bronchoalveolar lavage (BAL) from a gnotobiotic calf, six days after inoculation with the Snook strain of BRSV [30], which had previously been passaged twice in gnotobiotic calves [29]. The BAL was free from BVDV, mycoplasmas, and bacteria as assessed by inoculation of tissue culture or mycoplasmal or bacterial media (data not shown).

Virus titers were determined by plaque assay on fetal calf kidney (CK) cells or Vero cell monolayers in 35-mm-diameter petri dishes as described previously [26].

### Subunit Vaccine Production and Formulation

#### Construction, expression and purification of recombinant RSV proteins

The plasmids pGEX-M2-1, pGEX-P and pGEX-PCT (coding for residues 161–241 of the phosphoprotein (PCT)) derived from the pGEX-4T3 expression vector (Pharmacia), and pET-N derived from pET28a(+) vector (Novagen) plasmids which contain sequences from the HRSV Long strain have been described previously [31]–[33]. *E*. coli BL21(DE3) (Novagen) cells were transformed with pGEX- or pET-derived plasmids for expression of recombinant proteins (Table 1), as previously described [31]–[34]. The co-expression and co-purification of pET-N and pGEX-PCT to produce N nanorings (N^SRS^) composed of 10 or 11 protomers, has been previously described [32], [35]. For insertion of the F and G epitopes of BRSV strain 9402022 [36] into N to create epitope-decorated nanorings (eN; Table 1), complementary oligonucleotides coding for either: i) an F mimotope (HWSISKPQ) [37], ii) F residues 255–278 (SELLSLINDMPITNDQKKLMSSNV) [38], iii) F residues 422–438 (CTASNKNRGIIKTFSNG) [38], or iv) G residues 174–187 (STCEGNLACLSLCQ) [39], were annealed and inserted at *Age*I-*Xho*I sites (for fusion at the C-terminus of N) or *Nde*I-*Bam*HI sites (for fusion at the N-terminus of N) in the pET-N-GFP [34] and pET-N plasmids, respectively. Expression and purification of eN was performed as described for N^SRS^. All constructs were verified by sequencing. Proteins were quantified by absorption at 280****nm, except for eN proteins, which contain RNA and were quantified by the Bradford method.

**Table 1:** RSV proteins and epitopes used in subunit vaccine formulation.

Proteinproduct^a^	Description	Sequenceorigin
M2-1	Full length M2-1 protein^c^	HRSV^b^
P	Full length P protein^d^	HRSV^b^
eN-F_255–278_	Residues 255–278 of F at N terminus of N in eN^f^ (AA: SELLSLINDMPITNDQKKLMSSNV)	BRSV^e^
eN-F_422–438_	F residues 422–438 at C terminus of N in eN^f^ (AA: CTASNKNRGIIKTFSNG)	BRSV^e^
eN-F_mimo_	Residues mimicking epitope on F^g^ at C terminus of N in eN^f^ (AA: HWSISKPQ)	Combinatorial peptide
eN-G_174–187_	G residues 174–187 at N terminus of N in eN^f^ (AA: STCEGNLACLSLCQ)	BRSV^e^

a Protein product included in subunit vaccine formulations, as abbreviated in the current paper.

b HRSV Long strain (GenBank accession no. AY911262) was used to construct recombinant protein.

c Full length HRSV M2-1 protein (Long strain).

d Full length HRSV P protein (Long strain).

e BRSV strain 9402022 (Larsen et al. 1998) was used to construct recombinant protein.

f Selected residues were recombinantly attached to the N or C terminus of HRSV N protein (Long strain) and co-expresses with a fragment of HRSV P protein (Long strain, AA residues 161–241) to form N-nanorings with attached epitopes (eN) on each of 10 or 11 protomers.

g Antigenic site II (AA residues 422–438) on F, as described by Chargelegue et al. (1998).

#### Subunit vaccine formulation

Each dose of subunit BRSV vaccine contained 25** µ**g of each of the recombinant proteins (Table 1) in PBS, which were either: for the subunit Montanide vaccine (SUMont), mixed and emulsified with 1.4****ml of Montanide ISA71^VG^ (SEPPIC, France) in a 3∶7 (aqueous:oil) ratio, and a final volume of 2****ml; or for the subunit AbISCO vaccine (SUAbis), mixed with 144** µ**l (390** µ**g) AbISCO-300 (Novavax, Sweden), and diluted in PBS to a final volume of 2****ml. Each dose of placebo vaccine consisted of 390** µ**g AbISCO-300 diluted in PBS to a final volume of 2****ml.

### Calves and Experimental Design

**Figure 1:**
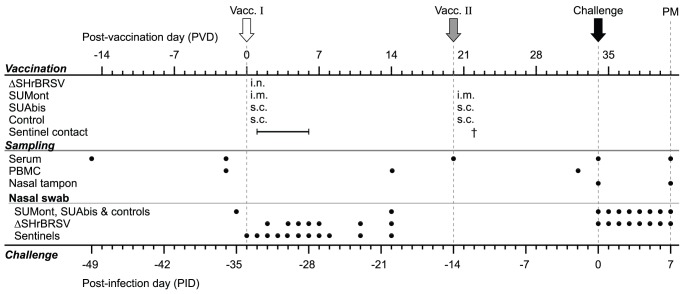
Experiment timeline, vaccination and sampling. Twenty calves with moderate titers of BRSV-specific serum antibodies (MDA) were allocated into 4 groups and vaccinated as indicated in the figure; all were vaccinated on post-vaccination day (PVD) 0 (Vacc. I, white arrow) with either (a) 5×10^6^ pfu of ΔSHrBRSV intranasally (i.n.); (b) BRSV and HRSV recombinant protein subunits (SU) adjuvanted by Montanide (SUMont) intramuscularly (i.m.), (c) SU adjuvanted by AbISCO-300 (SUAbis) subcutaneously (s.c.), or (d) adjuvant alone s.c. (Controls). On PVD 20, all animals except those immunized with ΔSHrBRSV, were boosted with the same formulation and route as for Vacc. I (Vacc. II, gray arrow). Three BRSV-seronegative calves were housed in contact with ΔSHrBRSV-infected animals to determine transmission of the vaccine virus (Sentinel calves), and monitored until euthanized (†) on PVD 22. On PVD 20, one calf in group c was euthanized due to traumatic injury. On post-infection day (PID) 0, all calves were challenged i.n. with 10^4^ pfu virulent BRSV (black arrow), and clinically scored daily until PID 7. Throughout the experiment, samples were collected, as indicated in the figure, to analyze antibodies in serum and nasal secretions, *ex-vivo* response of peripheral blood mononuclear cells (PBMC) to restimulation with BRSV, and virus shedding in nasal secretions (Nasal swab). At post-mortem examination (PM), lung lesions were recorded and tissue samples collected, as well as bronchoalveolar lavage (BAL) samples for antibody, BRSV RT-PCR and virus isolation.

Twenty-three conventionally-reared bull calves of Swedish Holstein and Swedish red and white breed were obtained from the certified BVDV-free Swedish Livestock Research Centre (Lövsta, Sweden). Natural infections of BRSV in the herd were ruled out by continuous seromonitoring of 5 seronegative animals for BRSV-specific IgG_1_ antibodies, for 2 months prior to the experiment. Twenty healthy calves were allocated into 4 groups of 5 calves based on their age and titers of BRSV-specific MDA (Table 2). In addition, 3 BRSV-seronegative calves were selected to act as sentinels to study the potential transmission of ΔSHrBRSV. After arrival at the animal facility, during the week of acclimatization, all the calves were treated with procaine benzyl penicillin (20 mg/kg/day intramuscularly) for five days. Each calf was vaccinated for the first time 34 days before the day of experimental BRSV challenge (PID 0), with either: (a) 5×10^6^ pfu of ΔSHrBRSV vaccine i.n. in a volume of 6 ml DMEM; (b) 2 ml of the SUMont vaccine i.m.; (c) 2 ml of the SUAbis vaccine s.c.; or (d) 2 ml placebo s.c. (Fig. 1). Approval to use a genetically modified microorganism within the context of this experiment was obtained from the Swedish Work Environment Authority (registration number 202100-1868 v8a1). Three weeks later, group b, c and d were boosted with the same formulation and route as in the first immunization (Fig. 1). Clinical signs including post-vaccination swellings at injection sites in the calves immunized with SU or adjuvant alone, classified as mild (<5×5 cm), moderate (<10×10 cm), marked (<15×15 cm) or severe (>15×15 cm), were monitored. The calves immunized with ΔSHrBRSV, along with the 3 in-contact sentinel calves, were kept in an isolated unit in the animal facility until three weeks after first vaccination, while the other groups were kept in separate rooms in another unit. The two units had separate ventilation and staff, and showers were required to exit each unit. To avoid direct contamination of sentinels with inoculum virus, sentinel calves were housed in a separate room until one day after vaccination, when 2 out of 3 were transferred to the room (20 m^2^) housing the ΔSHrBRSV-vaccinated calves, while 2 out of 5 ΔSHrBRSV-vaccinated calves were transferred to the room (20 m^2^) of the remaining sentinel calf. Co-housing lasted for six days, then the sentinel calves were again isolated from the ΔSHrBRSV-vaccinated calves, to avoid potential reinfection of the ΔSHrBRSV-vaccinated calves. The sentinel calves were clinically, immunologically and virologically monitored for two additional weeks (Fig. 1). Three weeks after inoculation with ΔSHrBRSV, calves were moved to a separate room in the same unit as the other calves in the animal facility. One calf vaccinated once with SUAbis (calf c5) 3 weeks previously, was euthanized for welfare concerns caused by a traumatic leg injury. Five weeks after the initial vaccination, calves were challenged by aerosolization [12] of 10^4^ pfu BRSV strain Snook in 4 ml BAL, diluted up to 5 ml in DMEM. Throughout the experiment, each calf was examined clinically on a daily basis and scored as previously described [12].

**Table 2:** Vaccine effect on virus load, clinical disease and lung pathology after BRSV challenge.

Immunization^a^	Calf	Age(weeks)^b^	MDA (log_10_titer^−1^)^c^	BRSV RT-PCR(log_10_ TCID_50_eq.)^d^		Virus isolatedfrom BAL(passage)^e^	Accumulatedclinical score^f^	Extent ofmacroscopiclesions (%)^g^
				Accum. NS	BAL			
ΔSHrBRSV once i.n.	a1	9	2.0	0.0	2.1	–	0	4
	a2	8	2.2	0.0	2.2	–	3	10
	a3	7	2.0	1.0	3.0	–	8	4
	a4	6	3.0	0.0	1.4	–	1	1
	a5	4	2.2	1.8	1.9	–	7	1
SUMont twice i.m.	b1	10	1.9	0.0	3.0	–	10	4
	b2	8	1.9	1.1	3.5	–	43	25
	b3	6	2.9	2.3	4.4	+P3	9	13
	b4	3	3.3	1.0	3.9	–	17	1
	b5	3	2.1	0.0	1.0	–	13	1
SUAbis twice s.c.	c1	9	2.2	2.8	4.5	+P3	48	22
	c2	8	1.9	1.6	4.3	+P3	28	18
	c3	8	2.4	2.7	4.3	+P3	12	11
	c4	6	2.0	1.4	3.8	–	37	32
	c5	2	2.3	†	†	†	†	†
Control twice s.c.	d1	11	1.9	3.0	4.2	+P1	70	64
	d2	8	2.6	2.0	5.0	+P1	27	36
	d3	6	2.1	3.0	5.8	+P1	106	38
	d4	4	2.9	2.9	4.5	+P2	78	45
	d5	2	2.3	2.6	5.3	+P1	86	57

a Conventional colostrum-fed calves were immunized with either ΔSHrBRSV, SUMont, SUAbis or adjuvant alone, as indicated in the table. All animals were immunized on post-infection day (PID) −34, and all animals except those immunized with ΔSHrBRSV were immunized again on PID −14, and all were challenged with BRSV on PID 0, as indicated in Figure 1.

b Age at first vaccination (weeks).

c Serum titer of BRSV-specific maternal antibodies (MDA) before first vaccination (PID −36) as determined ELISA.

d Accumulated viral-shed in nasal secretions and virus RNA detected in 1 ml of bronchoalveolar lavage (BAL) collected as indicated in figure 1. Samples were analyzed using BRSV-F real-time PCR after total RNA extraction. The unit TCID_50_ equivalent is determined by including standard dilution series of virus with a known TCID_50_ in the real-time PCR analysis.

e Virus isolated from post-mortem BAL cell samples, after inoculation of bovine turbinate epithelium cells in 25 cm^2^ flasks in three consecutive passages. In any of the three passages, cell cultures with evident cytopathic effects within seven days of culture were determined to be positive for BRSV in passage one (+P1), two (+P2) or three (+P3). Samples that were negative after three passages are noted as negative (–).

f Accumulated clinical score, calculated as the area under the curve of daily clinical scores, in turn determined by clinical examination.

g Extent of lung lesions were determined post mortem and are presented as a percentage of lesioned lung tissue per calf.

† Calf c5 was euthanized before challenge due to traumatic injury.

### Sampling

Heparinized and unmodified venous blood samples were collected from all calves as indicated in Figure 1. Peripheral blood mononuclear cells (PBMC) and serum were extracted from heparinized and unmodified blood, respectively. PBMCs were used directly in lymphocyte proliferation assays, whereas serum was stored at −20°C.

Nasal secretions were collected as indicated in Figure 1, and stored at −70°C, as previously described [12] using sterile cotton-tipped swabs (NS) and tampons (NT). On PID 7, the calves were euthanized by an overdose of general anesthesia (5 mg/kg ketamine and 15 mg/kg pentobarbital sodium) followed by exsanguination.

Lungs were excised and lesions photographed, palpated and recorded on a standardized chart; after scanning and digitalization, the proportion of consolidated lung parenchyma was calculated as a percentage of the total lung area (Adobe Illustrator CS5, version 15.1 for Mac). BAL samples were collected post-mortem from the left lung of all challenged calves, as previously described [10]. After aliquoting, BAL cells corresponding to 10 ml of BAL fluid were pelleted by centrifugation (200×g, 10 min), counted manually in a Bürker chamber, and resuspended in 350 µl RLT buffer (Qiagen) or 1 ml DMEM containing 20% fetal calf serum and stored at −70°C, along with the recovered supernatant, for further analysis. Samples of lung tissue were collected post-mortem, preferentially from consolidated areas, of each of the lobes in the right lung (cranial part of cranial lobe, caudal part of cranial lobe, caudal lobe and accessory lobe), fixed in 10% buffered formalin, embedded in paraffin and sectioned.

### Detection of BRSV

#### Detection of BRSV RNA

BRSV RNA coding for the F protein present in nasal secretions or in BAL cells corresponding to 10****ml of BAL, was quantified by RT-PCR as previously described [12]. The unit TCID_50_ equivalent (TCID_50_ eq.) was used since the standard curve in this assay was based on a BRSV infected cell lysate with a known titer (10^5.8^ TCID_50_).

#### Isolation of BRSV from BAL cells

Isolation of BRSV present in BAL cell samples was attempted by inoculating 95% confluent BT cells in 25****cm^2^ tissue culture flasks, as described previously [12]. Inoculated cells were examined daily for seven consecutive days for cytopathic effects. Supernatants from samples not showing cytopathic effects were passed a further two times in new 25****cm^2^ flasks.

### Humoral Responses

#### ELISA for detection of BRSV- and protein-specific IgG and IgA

BRSV-specific IgG_1_ antibody titers were determined using a commercially available kit (SVANOVIR BRSV-Ab ELISA, Boehringer Ingelheim Svanova, Sweden) in accordance with the manufacturer’s instructions. HRSV-F, -N, -P and -M2-1 IgG antibodies were analyzed as described previously for N^SRS^ [25], using ELISAs based on the relevant purified protein described herein for N, P and M2-1, and previously for F [40]. BRSV-specific IgA antibodies were detected by capture ELISA, as previously described by Uttenthal et al. 2000 [41] and modified as earlier reported [12]. RSV-N-specific IgA antibodies were detected using N^SRS^-coated microtiter plates as previously described [34], with the following alterations: blocking after coating was achieved by 1h incubation with 2% BSA in PBS; mouse anti-bovine IgA (Serotec, MCA2438) was used to detect IgA in samples; and rat anti-mouse IgG_2a_ conjugated to HRP (Serotec, MCA1588P) was used to elucidate anti-IgA. BRSV-G specific IgG antibodies were determined by ELISA as previously described [15] using a lysate of chick embryo fibroblasts (CEF) infected with recombinant fowlpox virus (rFPV) expressing the G protein of the Snook strain of BRSV, produced as described previously for rFPV expressing other BRSV proteins [14]. A lysate of CEF infected with wild-type FPV was used as a control. Sera were serially diluted and end-point titers calculated from corrected optical density (COD), as previously described [12]. Nasal secretions were not titrated, but instead diluted ∶20 and the level of BRSV-specific IgA expressed as percentage of a positive control sample.

#### Virus neutralizing antibody assay

Neutralizing antibodies to BRSV in heat-inactivated serum were determined by a plaque reduction assay on fetal calf kidney cells as described previously [42].

### Cellular Responses

#### BRSV-specific lymphocyte proliferation assay

Peripheral blood mononuclear cells (PBMC) were isolated from heparinized blood of all animals, as described previously [10]. Additionally, tracheobronchial lymph nodes (LN) of ΔSHrBRSV-immunized and control calves, collected on PID 7, were mechanically disrupted and lymphocytes were isolated by centrifugation over Ficoll Paque Plus (GE Healthcare) during 8****minutes at 800×*g*. PBMC and lymph node cells were restimulated with heat-inactivated (56°C for 30****min) BRSV-infected (no. 9402022, Denmark [43]) or uninfected BT cell lysate. After 7 days of incubation, Alamar Blue (Invitrogen, Sweden) was added and, following an additional 8 (PBMC) or 24 (LN cells) hours of incubation, optical absorbance (OD) was measured at 570****nm and 595****nm (Multiskan EX 355, Thermo Fisher Scientific, USA) and adjusted according to the manufacturer’s instruction. Corrected OD (COD) was calculated by subtracting OD_595nm_ from OD_570nm_, and then OD_control_ from OD_BRSV_. After determination of proliferative response to restimulation and following centrifugation at 200×*g*, supernatants were recovered and stored at **−**80°C for cytokine analysis.

#### Flow cytometric analysis of BRSV-specific IFNγ-producing lymphocytes

Tracheobronchial LN cells were restimulated as above, in duplicates of 5×10^6^ cells/calf for 18 hours. Brefeldin A (Sigma) was added for the last 15 hours at 10** µ**g/ml. Cells were stained for viability (*LIVE*/*DEAD* Fixable *Near*-*IR* Dead Cell Stain, Life Technologies) and for expression of surface markers, CD4 and CD8 (MCA1653F:FITC (CD4), MCA837A647: AlexaFlour 647 (CD8), AbD Serotec). Cells were then fixed for 10****min with 4% (w/v) paraformaldehyde in PBS, and cell membranes were permeabilized (FACS permeabilization solution 2, BD Biosciences) prior to intracellular staining for IFNγ (MCA1783PE: RPE (IFNγ), AbD Serotec). Cells were assayed using a flow cytometer (FACSVerse, BD Biosciences) and data were analyzed using FACSuite software. Non-aggregating, live cells (3300–20000, mean 17500) were gated based on light-scattering properties and fluorescence at 783/56****nm. Gates for CD8, CD4 and IFNγ were set based on Fluorescence Minus One controls.

#### ELISA for detection of bovine IL-4 and IFNγ

Bovine interleukin-4 (IL-4) and interferon gamma (IFNγ) were detected in supernatant from restimulated lymphocytes using commercially available kits (Bovine IL-4 ELISA, MCA5892KZZ, and Bovine IFNγ ELISA, MCA5638KZZ, Bio-Rad), in accordance with the manufacturer’s instructions. Concentrations were derived by including dilution series of supplied standard samples of recombinant protein, and expressed as ng/ml.

### Histology

Histological sections of lung tissue were stained with hematoxylin and eosin (HE) or carbol chromotrope (CC) histochemical stain to demonstrate eosinophils, and were evaluated in a blinded manner. Cell subpopulation characteristics and any inflammation in each section was morphologically described and scored as either normal (0), mild (1), moderate (2) or severe (3), as previously described [12]. Individual severity of histopathology in consolidated areas was calculated as the mean score of all sections (described above) per calf.

### Data Analysis

#### Statistical analysis

Statistically significant differences between groups, with regard to each set of collected and aggregate data, was determined using either one-way ANOVA followed by Student’s *t*-test, or Kruskal–Wallis analysis followed by Wilcoxon test (JMP 10 for Mac, SAS Institute Inc.), if not otherwise specified. Significance was assumed when p≤0.05 (*), p≤0.01 (**), p≤0.005 (***) or p≤0.001 (****).

#### Accumulated clinical score and viral shedding

Accumulated clinical score (ACS) and accumulated viral shedding (AVS) from PID 0 to PID 7 was calculated as the area under curves using the trapezoidal rule.

#### Ranking

The ACS and AVS represent the sum of individual clinical disease and viral load, respectively, regardless of the time they occurred during the challenge experiment. Likewise, the proportion of consolidated lung tissue post-mortem represents accumulated lung injury. Based on these criteria, three ranks were constructed and all calves were ranked (1–19) to indicate the individual level of disease and viral replication following challenge. The highest rank (19) was assigned to the most affected calf, and in decreasing order to the least affected, with a high clinical rank indicating a high accumulated clinical score (ACS); a high lung lesion rank indicating a high percent of macroscopic lung lesions post-mortem; a high viral-shed rank indicating a high accumulated viral-shed (AVS). Statistical differences between groups were calculated using individual rank sum, defined as the sum of these three ranks for each calf. In addition, group rank sums were calculated for each of the three ranks and corrected for number of calves per group (n), since one of the calves (d5) in the SUAbis-vaccinated group was excluded, by division by n followed by multiplication by 5, and a total rank sum calculated.

## Results

### Post Vaccination Monitoring

#### Intranasally administered ΔSHrBRSV appeared to be clinically safe and did not transmit to sentinels

Following i.n. vaccination with ΔSHrBRSV, only very slight upper respiratory signs (e.g. slight nasal discharge and infrequent coughing) were irregularly observed in all animals, in the week following vaccination. Marginal amounts of viral RNA were detected in nasal secretions of only one of these calves (calf a2; ≤0.36 TCID_50_ eq. unit; 5–7 days post-vaccination). In the 3 seronegative sentinel calves housed with the ΔSHrBRSV calves, clinical signs of respiratory disease were not observed, and virus was not detected in nasal secretions by RT-PCR (data not shown). Furthermore, an increase in BRSV-specific serum IgG_1_ was not detected in sentinel animals, three weeks after first contact with ΔSHrBRSV-vaccinated calves (data not shown). The absence of clinical signs, viral shedding and seroconversion indicated that transmission of ΔSHrBRSV to the sentinels had not occurred.

#### Vaccination by parenteral route with SUMont, SUAbis and AbISCO-300 alone generated transient local and general adverse reactions

Following the first vaccination with SUMont, all the calves were slightly depressed and developed elevated rectal temperatures (mean max ± SD 40.8±0.29°C) for 2–3 days post-vaccination, but no or only mild diffuse swelling was seen at the injection sites. The SUMont-vaccinated animal’s reaction to the second vaccination was similar (mean max ± SD rectal temperature 40.0±0.11°C), except for moderate to marked swelling at injection sites.

Calves immunized with SUAbis had elevated rectal temperatures following the first vaccination (mean max ± SD 40.3±0.19°C), with mild to moderate swellings at injection sites, which waned within 2–3 days. Following the vaccination boost, no or mild to moderate swellings were seen, but calves did not develop significantly elevated rectal temperatures. The general and local clinical signs observed in calves immunized with AbISCO-300 alone were similar to those described for SUAbis, except for one control calf (calf d5) which demonstrated elevated rectal temperatures following both first and second vaccination (peaked at 39.5°C and 40.1°C, respectively).

### ΔSHrBRSV and SUMont Induced Strong and Good Clinical Protection, Respectively, Against Virulent BRSV Challenge

**Figure 2:**
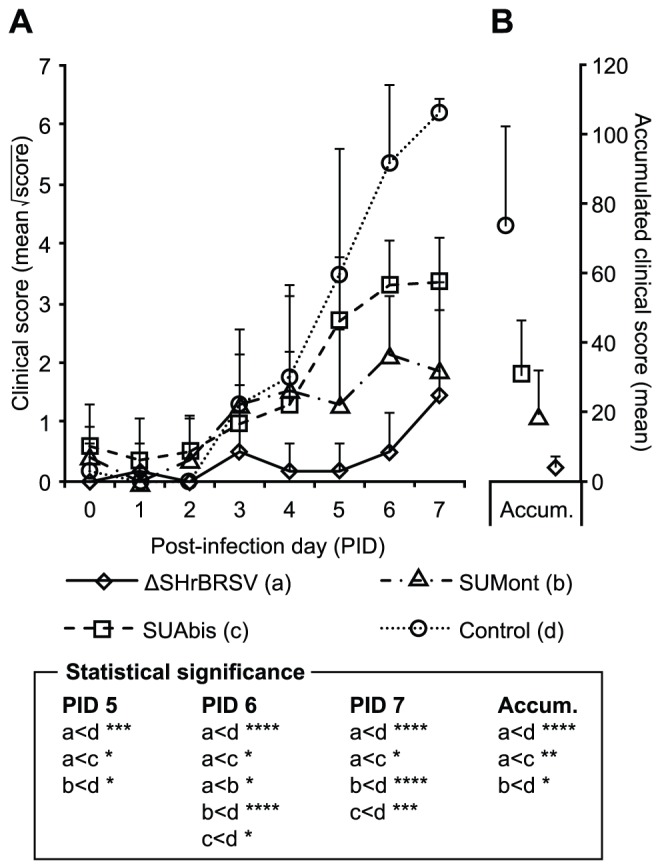
Vaccination protects against clinical signs of BRSV disease. Four groups of 5 calves were vaccinated as described in Fig. 1 and challenged with BRSV, 5 weeks after vaccination, on post-infection day (PID) 0. Following challenge, calves were examined daily until euthanization on PID 7, and the severity of clinical signs of diseases were scored as previously described [12]. (A) presents the mean square root of clinical scores per day (to approximate normal distribution for statistical analysis), and (B) the accumulated clinical score from PID 0 to PID 7, with standard deviations indicated by upward deflecting lines. Statistically significant differences are indicated by asterisks p≤0.05 (*); p≤0.01 (**); p≤0.005 (***); p≤0.001 (****); p≤0.0001 (*****).

Despite moderate titers of MDA at the time of challenge, control calves developed marked to severe clinical signs as previously observed in this model [12] (Blodorn et al, in preparation). The clinical scores of the control calves presented in figure 2A reflect the progression from mild respiratory signs appearing on PID 3, to severe upper and lower respiratory disease on PID 7. Severe signs included a severely depressed general state and fever (max 40.2–41.2°C mean max 40.8°C); markedly reduced or absent appetite; coughing; nasal discharge; moderate to severe abdominal dyspnea; wheezing lung sounds on auscultation and increased respiratory rate (mean max 81.6 SD±4.6 breaths/min). One control (calf d3), reached the end-point at the time of euthanization on PID7. The mean accumulated clinical score from PID 0 to PID 7 (ACS) of the control group was 73.2 (SD±29.1; Fig. 2B).

In contrast to the controls, all vaccinated animals exhibited varying degrees of clinical protection (Fig. 2A–B). Clearly, the most clinically protected were animals immunized once i.n. with ΔSHrBRSV, with a mean ACS of 3.7 (SD±3.6), which was significantly lower than that of control calves (p≤0.001) or animals immunized twice with SUAbis (p≤0.05), and also tended to be lower than those immunized twice with SUMont (p = 0.06; Fig. 2B). Throughout the challenge, none of the ΔSHrBRSV-vaccinated calves had a reduced appetite or depressed general state. Apart from one calf (a1), which showed no clinical signs, and one calf (a5), which exhibited mild dyspnea and slight wheezing lung sounds on PID 7, calves immunized with ΔSHrBRSV showed very mild signs of respiratory disease. These mild signs were recorded on the last two days of the challenge (PID 6 and PID 7), and included slight serous nasal discharge, coughing on provocation, and slightly enhanced lung sounds. From PID 0 to 7, only one of the ΔSHrBRSV immunized calves had a peak rectal temperature exceeding 39.5°C (calf a2, 39.6°C on PID 7), and none had more than slightly elevated respiratory rate (mean max 49.6 SD±3.6 breaths/min).

Compared to controls, significant clinical protection was also observed in calves immunized with either of the subunit vaccines (Fig. 2A–B). In calves immunized with SUMont, respiratory signs were first observed on PID3, and peaked at moderate levels on PID6, when the group mean clinical score was significantly higher than that of ΔSHrBRSV (p≤0.05; Fig. 2A). These mild and moderate clinical signs included serous nasal discharge, spontaneous coughing, slight to moderate dyspnea, slight wheezing lung sounds and elevated respiratory rate (mean max 54.4 SD±8.3 breaths/min). From PID 0 to PID 7, only 2/5 calves immunized with SUMont demonstrated rectal temperatures above 39.5°C (calf b2, 39.9°C on PID 4; calf b4, 39.7°C on PID 2). Compared to that of controls, the level of clinical disease observed in the SUMont calves was significantly lower on PID 5 (p≤0.05), PID 6 (p≤0.001) and PID 7 (p≤0.001), yielding a highly significantly lower ACS (mean ± SD 18.0±14.0, p≤0.005) (Fig. 2 and Table 2A–B).

Although the calves immunized with SUAbis were afforded clinical protection compared to controls on PID 6 (p≤0.05) and 7 (p≤0.005), they were less protected than calves vaccinated with SUMont (p = 0.26), and significantly less protected than calves vaccinated with ΔSHrBRSV (p≤0.05), in terms of ACS (mean ± SD 31.0±15.0; Fig. 2A–B). The severity of clinical signs observed in calves immunized with SUAbis was intermediate to those observed in calves vaccinated with SUMont and control animals (Fig. 2A–B), with a mean peak respiratory rate of 61.0 breaths/min (SD±6.8 breaths/min), and with 3/4 calves having peak rectal temperatures over 39.5°C after challenge (calf c1, 40.2°C on PID 6; calf c2, 39.7°C on PID 3; calf c4, 39.7°C on PID 7).

In summary, whereas a single i.n. administration of ΔSHrBRSV in calves with moderate to high titers of BRSV-specific MDA induced almost complete clinical protection against virulent challenge, two parenteral administrations of SUMont induced a good level of clinical protection, while SUAbis afforded some clinical protection, compared to controls (Table 2, Fig. 2A–B).

### ΔSHrBRSV and SUMont Afforded Almost Complete and Good Protection, Respectively, Against Pathologic Changes in the Lungs

**Figure 3:**
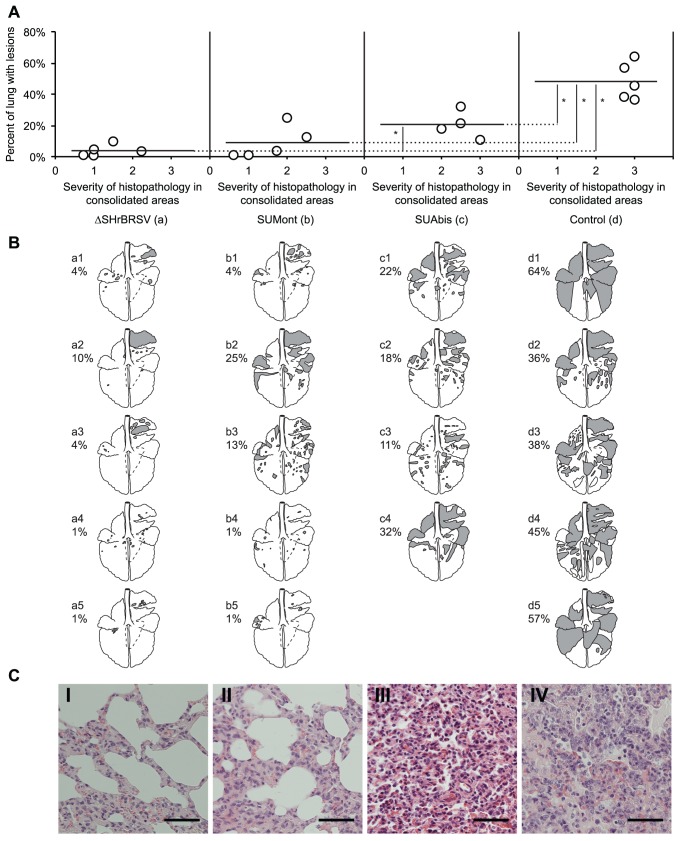
Vaccination reduces the extent of lung lesions following BRSV challenge. Four groups of 5 calves were vaccinated as described in Fig. 1 and challenged with BRSV, 5 weeks after vaccination. Two weeks before challenge, one calf (c5) was euthanized due to traumatic injury. Lungs were removed after exsanguination, lesions were recorded on a lung chart after visual examination and palpation, and the proportion of lung showing pneumonic consolidation was calculated. Formalin-fixed tissue samples from each lobe in the right lung were analyzed for the severity of histopathological changes and scored as either normal (0), mild (1), moderate (2) or severe (3). (A) shows the extent of macroscopic lesions on the y-axes, and the microscopic severity of inflammation (mean score of four sections per calf) on the x-axes. Statistically significant difference is indicated by asterisks (p≤0.05). (B) shows the percent of pneumonic consolidation in each animal (also depicted as filled areas in lung-charts), and emphysema (outlined areas in calves d3 and d4). Panels C (I–IV) show representative histological images from each of the four groups of calves. Bar indicate 100 µm. Panels C (I) (ΔSHrBRSV), C (II) (SUMont), C (III) (SUAbis) and C (IV) (Control) show lung parenchyma with minimal, mild, moderate and severe pathological changes, respectively.

The pathological observations were well in agreement with the clinical data. Whereas a high percentage of gross pneumonic consolidation was observed in controls at necropsy, 7 days after challenge, significantly less macroscopic lesions were observed in the lungs of all vaccinated calves (p≤0.05; Fig. 3A). Among the vaccinated animals, the ΔSHrBRSV-vaccinated calves had less lesions, compared to the SUMont-vaccinated calves, but the difference was not statistically significant (p = 0.66). Furthermore, while calves immunized with either ΔSHrBRSV or SUMont had less lesions compared to those immunized with SUAbis (Fig. 3A), this was only statistically significant for ΔSHrBRSV (p≤0.05). Overall, the majority of lesions were in the cranial parts of the lungs, but the extent of consolidated tissue varied from small and scattered lesions in the animals immunized with ΔSHrBRSV, to massive areas of consolidation involving almost half the lungs in the controls (Fig. 3B). Apart from areas of consolidation, two calves (d3 and d4) in the control group also exhibited moderate lung pleural emphysema.

Tissue from consolidated areas of the lungs from all calves were histologically examined, and proliferative and exudative bronchiolitis with accompanying alveolar collapse and peribronchiolar infiltration by mononuclear cells was observed, as previously described for BRSV-infection in calves [44]. These lesions were severe in control animals, with decreasing intensity in SUAbis-, SUMont- and ΔSHrBRSV-immunized animals, in that order.

Lesions were most pronounced in sections from the cranial lobes, whereas the caudal and accessory lobes were less severely affected. Histological lesions were most marked in controls (score 2.8–3.0, mean 2.9), followed by animals immunized with SUAbis (score 2.0–3.0, mean 2.5), followed by animals immunized with SUMont (score 0.6–2.5, mean 1.6), and least severe in animals immunized with ΔSHrBRSV (score 0.8–2.3, mean 1.3) (Fig. 3A). Panels C (I–IV) in figure 3 show representative histological images from each of the vaccinated and unvaccinated groups of animals. Whereas panel C (I) shows lung parenchyma from a ΔSHrBRSV-immunized calf (a2), with minimal thickening of the alveolar walls, panel C (II) shows lung parenchyma with mild pathological changes from a SUMont-immunized calf (b3), with slight thickening of the alveolar walls, but where alveolar spaces are still clear. On the other hand, calves immunized with SUAbis (panel C (III), calf c2) demonstrated moderate pathological changes in the lung parenchyma, with moderate thickening of the alveolar walls, and mononuclear inflammatory cells and a few neutrophils in the alveolar spaces. In the unvaccinated control animals (panel C (IV), calf d1) severe pathological changes were evident in the lung parenchyma, with severe thickening of alveolar walls, and scattered type II-cells lining the alveoli. Furthermore, in these animals, alveolar spaces were filled with numerous mononuclear inflammatory cells, some neutrophils and occasional syncytial cells. For all calves, inflammatory cell infiltration consisted of mononuclear cells and neutrophils, with very few eosinophils.

BAL cells count showed that control animals had significantly more cells in BAL (p≤0.01; mean±SD 11.0±3.7 cells × 10^5^/ml), compared to animals immunized with ΔSHrBRSV (mean±SD 5.2±3.5 cells × 10^5^/ml), SUMont (mean±SD 3.1±1.8 cells × 10^5^/ml) and SUAbis (mean±SD 3.2±1.9 cells × 10^5^/ml).

In summary, among vaccinated calves, ΔSHrBRSV-immunized calves were best protected based on lung pathology after challenge, followed by calves immunized with SUMont, and the least protected SUAbis-immunized calves. There was no evidence of exacerbated pulmonary pathology in any of the vaccinated calves.

### Vaccine-induced Virological Protection Consistent with Clinical and Pathological Protection

**Figure 4:**
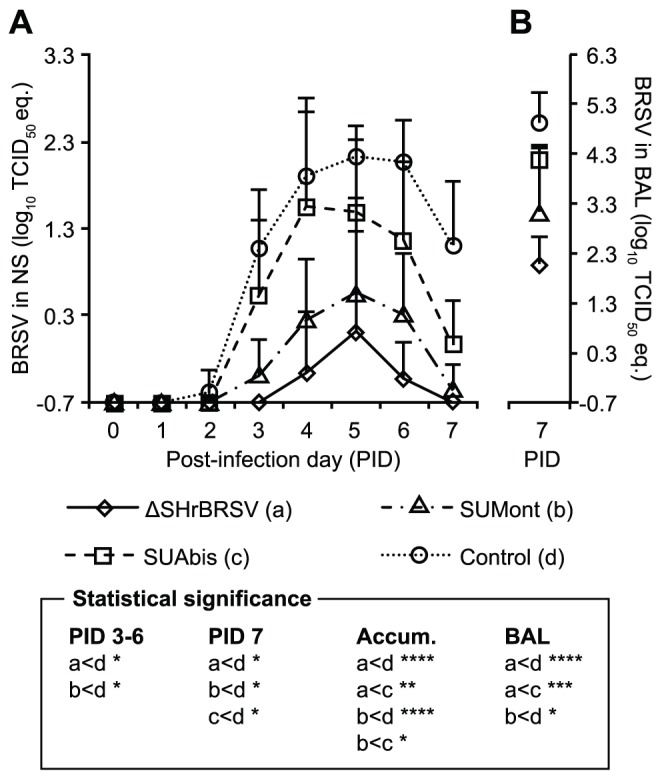
Vaccination reduces virus load in upper and lower airways following virulent BRSV challenge. Four groups of 5 calves were vaccinated as described in Fig. 1 and challenged with BRSV, 5 weeks after vaccination, on post-infection day (PID) 0. Two weeks before challenge, one calf (c5) was euthanized due to traumatic injury. The figure presents mean viral load in nasal swabs collected from PID 0 to PID 7 in panel A and post-mortem bronchoalveolar lavage (BAL) in panel B, as determined by BRSV F-gene RT-PCR after total RNA extraction, and is expressed as TCID_50_ equivalent, calculated from standard dilution series of virus with a known TCID_50_. The area under mean curves in panel A represents the accumulated detected virus shed (AVS): calves immunized with either ΔSHrBRSV or SUMont had significantly lower AVS (1.4±2.2 eqTCID_50_, p≤0.005 and 3.6±2.6 eqTCID_50_, p≤0.05 respectively), compared to calves immunized with either SUAbis (7.9±3.4 eqTCID_50_) or adjuvant alone (11.0±2.2 eqTCID_50_). Statistically significant difference with Student’s *t*-test are indicated by asterisks and the corresponding groups; p≤0.05 (*); p≤0.01 (**); p≤0.005 (***); p≤0.001 (****).

The extent of BRSV infection in the controls was demonstrated by high levels of BRSV RNA detected in nasal secretions of all control calves from PID 3 to PID 7, with a peak on PID 5 (mean ± SD 2.2±0.36 log_10_ TCID_50_ eq. unit; Fig. 4A). In the lower respiratory tract, high levels of viral RNA were detected in BAL cells from control calves, collected on PID7 (mean ± SD 5.0±0.62 log_10_ TCID_50_ eq. unit; Fig. 4B). Accordingly, live BRSV was isolated from the lower respiratory tract of the controls, after inoculation of BAL cells on cell cultures, followed by one or two passages (Table 2). The accumulated virus shed (AVS) in nasal secretions of the control calves was 11.0±2.2 log_10_ TCID_50_ eq. unit.

In contrast to the control calves, calves immunized with ΔSHrBRSV were very well protected against BRSV replication following challenge, since only low quantities of BRSV RNA were detected in nasal secretions (max mean ± SD 0.1±1.1 log_10_ TCID_50_ eq. unit; Fig. 4A, Table 2), and only in 2/5 calves (calves a3 and a5, Table 2) for 2 and 3 days respectively. Furthermore, the mean AVS in the upper respiratory tract for the group of calves immunized with ΔSHrBRSV (mean ± SD 1.4±2.2 log_10_ TCID_50_ eq. unit) was highly significantly reduced compared to controls (p≤0.0001), and the mean AVS of controls were 10^9^ times higher. In addition, only low quantities of BRSV RNA could be detected in the BAL collected on PID 7 (mean ± SD 2.1±0.6 log_10_ TCID_50_ eq. unit, Fig. 4) and virus could not be isolated in cell culture, even after three passages (Table 2).

Virologically, calves immunized with SUMont were also well protected, since BRSV RNA was only detectable in NS for 2–4 days (mean 3.4 days), amounting to a significantly lower AVS (mean ± SD 3.6±2.6 log_10_ TCID_50_ eq. unit; p≤0.001) compared to controls, which had a 10^7^ times higher AVS. Moreover, the BRSV RNA in BAL of SUMont animals was significantly reduced (p≤0.001), compared to controls (Fig. 4B), and virus was isolated only from one SUMont calf (calf b3, 3^rd^ passage; Table 2). However, compared to ΔSHrBRSV animals, the mean AVS of the SUMont-vaccinated animals was 10^2^ times greater, although this was not significant due to individual variation (p = 0.2).

Finally, calves immunized with SUAbis showed some degree of virological protection compared to controls, but less than that in vaccinated calves from other groups, since BRSV RNA could be detected in NS for 4–5 days (mean 4.5 days), with an AVS (mean ± SD 7.9±3.4 log_10_ TCID_50_ eq. unit) which was less than one-thousandth (1/10^3^) that of controls, but 10^6^ times higher than the ΔSHrBRSV calves (p≤0.005), and 10^4^ times higher than the SUMont calves (p≤0.05, Fig. 4A). The virological protection of the lower airways of the SUAbis calves was similarly intermediary, as less BRSV RNA was detected by RT-PCR in BAL samples from these calves, compared to the control calves, but more than in BAL from the other vaccinated groups (Fig. 4B). Furthermore, whereas infectious virus was isolated from the BAL cells of all control calves in passage one or two, virus could only be isolated from BAL cells from 3 out of 4 calves vaccinated with SUAbis, and only in passage three (Table 2).

In summary, the vaccine-induced virological protection was in accordance with the clinical and pathological protection observed. Calves immunized with i.n. ΔSHrBRSV, followed by calves immunized with i.m. SUMont, were better protected virologically in the upper and lower respiratory tracts against challenge with virulent BRSV, compared to animals immunized with SUAbis or adjuvant alone (Fig. 4A–B).

### Ranking

**Figure 5:**
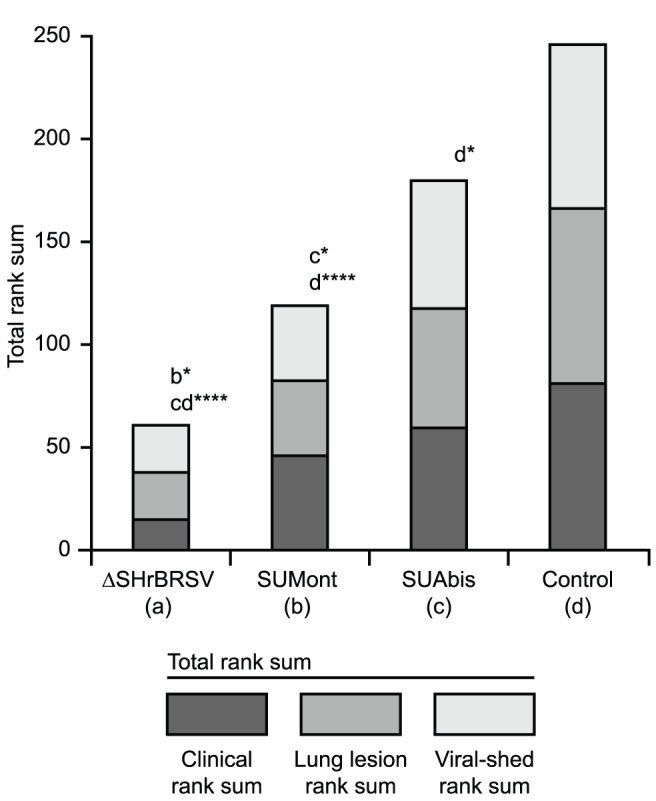
Vaccination reduces clinical signs, lung pathology and viral replication, following virulent BRSV challenge. Four groups of 5 calves were vaccinated as described in Fig. 1 and challenged with BRSV, 5 weeks after vaccination. Two weeks before challenge, one calf (c5) was euthanized due to traumatic injury. Calves were ranked (1–19) in each of three post-challenge parameters, with a high clinical rank indicating a high accumulated clinical score (Fig. 2); a high lung lesion rank indicating a high percent of macroscopic lung lesions post-mortem (Fig. 3); and a high viral-shed rank indicating a high accumulated viral-shed following challenge (Fig. 4). The figure shows the group sum of each of these ranks. To correct for the unequal number of calves per group (n), each rank sum was divided by n, and multiplied by 5. The stacked bars per group represent the sum of rank sums (total rank sum). Statistically significant differences in individual rank sums are indicated by asterisks and the corresponding group; p≤0.05 (*); p≤0.001 (****).

The three ranks: clinical rank, viral-shed rank and lung lesion rank; reflected the significant differences detected between groups in clinical signs, extent of lung lesions, and virus shed. Thus, the relative order of group rank sums was consistent across clinical, virological and pathological ranking, yielding consistent group total rank sums (Fig. 5): i) the ΔSHrBRSV-immunized animals were significantly protected compared to SUMont-immunized animals (p≤0.05), to SUAbis-immunized animals (p≤0.001) and to control animals (p≤0.001), ii) SUMont-vaccinated animals were significantly more protected than the SUAbis-vaccinated animals (p≤0.05) and controls (p≤0.001), and iii) SUAbis-immunized animals in turn, were significantly protected compared to controls (p≤0.05).

### Immunology

#### Systemic humoral immune responses

**Figure 6:**
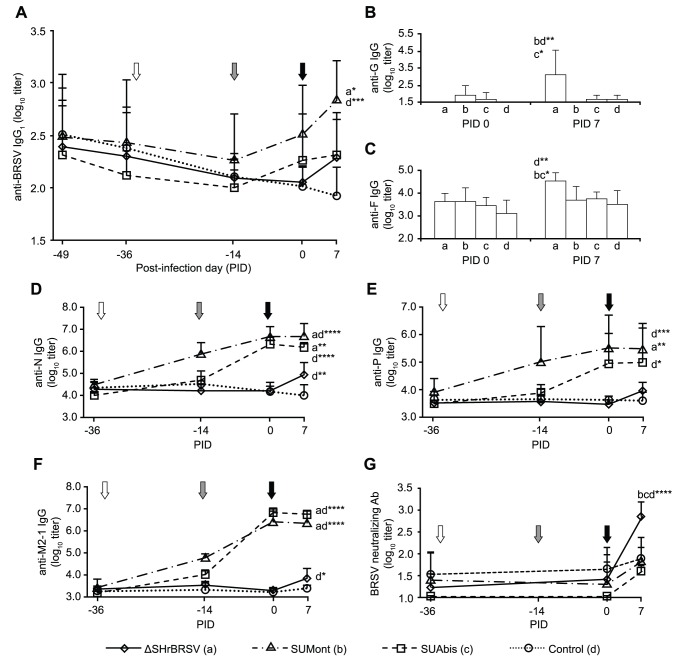
RSV-specific serum antibodies in calves before and after immunization and subsequent challenge with virulent BRSV. Four groups of 5 calves were vaccinated as described in Fig. 1 (white and grey arrows) and challenged with BRSV, 5 weeks after vaccination (black arrow) on post-infection day (PID) 0. Two weeks before challenge, one calf (C5) was euthanized due to traumatic injury. Panels show group mean log_10_ serum titers of: (A) BRSV-specific IgG_1_ (by ELISA); (B) IgG directed against BRSV G on PID 0 and PID 7 (by ELISA); (C) IgG directed against HRSV F on PID 0 and PID 7 (by ELISA); (D) IgG directed against HRSV N (by ELISA); (E) IgG directed against HRSV P (by ELISA); and (F) IgG directed against HRSV M2-1 (by ELISA) (G) BRSV-neutralizing antibodies (by plaque reduction assay). Note that the scale of the y-axis is not uniform between panels. Statistically significant difference on PID 7 is indicated by asterisks and the corresponding group; p≤0.05 (*); p≤0.01 (**); p≤0.005 (***); p≤0.001 (****).

Serum IgG antibodies against total BRSV; F, N, P, M2-1 of HRSV and G of BRSV were measured by ELISA. All calves except the sentinels had moderate to high, and statistically homogenous, titers of maternal BRSV-specific serum IgG_1_ antibodies at the time of first vaccination (Fig. 6A, Table 2). In the three weeks following first vaccination, BRSV-specific serum antibodies either continued to decline or remained unchanged (Fig. 6A). However, in the two weeks following the second vaccination, a slight increase in BRSV-specific serum antibody titers were observed in calves immunized with either SUMont or SUAbis, and these reached their highest levels one week after challenge, at the termination of the experiment. Following the second vaccination, the mean titer of BRSV-specific serum antibody titers in calves immunized with SUMont was consistently higher than that of calves immunized with SUAbis (Fig. 6A), but the difference was not statistically significant.

In the animals immunized once i.n. with ΔSHrBRSV, BRSV-specific IgG_1_ serum antibodies continued to decline after vaccination until one week after challenge, when they had rapidly increased (Fig. 6A).

The inhibitory effect of MDA on priming did not affect antibody responses to all BRSV proteins equally (Fig. 6A–G). Indeed, despite the apparent continued decrease of total BRSV-specific serum IgG_1_ (Fig. 6A), titers of IgG antibodies in serum directed against the N, P and M2-1 proteins in the SU, were already increasing after the first vaccination (Fig. 6D–F). In contrast, titers of F- and G-specific serum antibodies in SU-vaccinated animals were not significantly different compared to controls (Fig. 6B–C).

In calves immunized with ΔSHrBRSV, an increase in serum IgG antibodies against N, P, M2-1, G or F was not detected before challenge. However, one week after challenge, antibody titers specific to the F and G proteins were significantly higher in these animals, compared to animals in all other groups (p≤0.05; Fig. 6B–C). The ΔSHrBRSV animals also demonstrated a relative increase in antibody titers specific to N, P and M2-1 following challenge, compared to controls (p≤0.01, p = 0.45 and p≤0.05, respectively), but the antibody responses were less than those seen in animals vaccinated with SU (Fig. 6D–F).

BRSV neutralizing antibodies were also quantified in sera. Before challenge, no significant increase in neutralizing antibodies was detected in sera from any of the vaccinated calves (Fig. 6G). However, following challenge, ΔSHrBRSV-vaccinated calves demonstrated a significant increase in neutralizing antibodies (p≤0.001), and had significantly higher titers on PID 7, compared to all other animals (p≤0.001; Fig. 6D).

#### Local humoral immune responses

**Figure 7:**
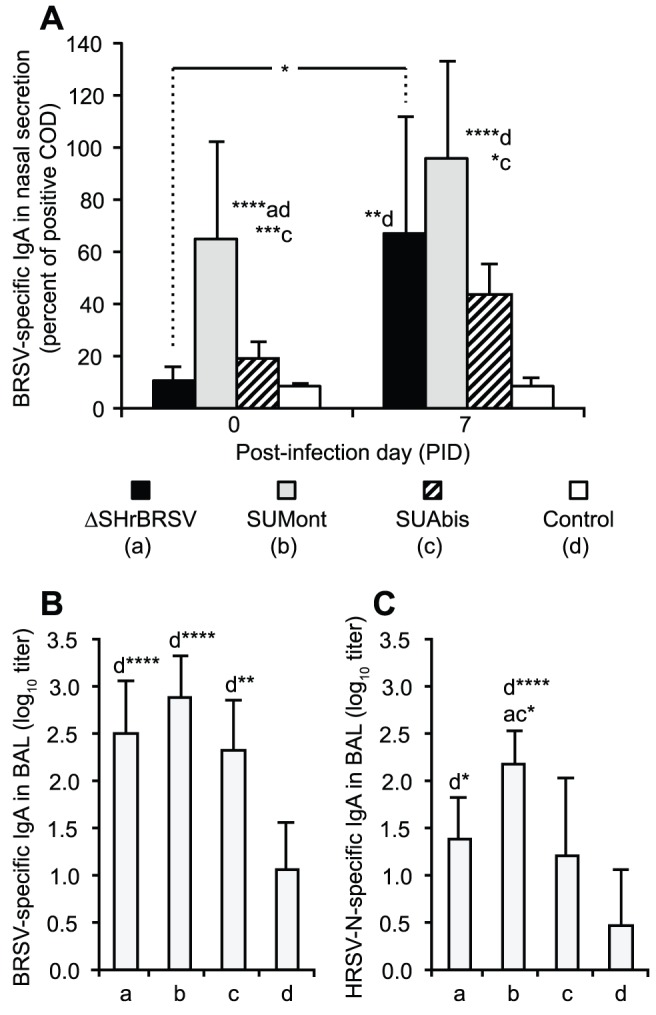
Mucosal IgA antibodies in the upper and lower airways, before and after BRSV challenge. Four groups of 5 calves were vaccinated as described in Fig. 1 and challenged with BRSV, 5 weeks after vaccination. Two weeks before challenge, one calf (c5) was euthanized due to traumatic injury. BRSV-specific IgA antibodies were analyzed by ELISA. (A) shows group mean levels of BRSV-specific IgA in nasal secretions on post-infection day (PID) 0 and 7, whereas (B) and (C) show group mean titers of total BRSV- and HRSV-N-specific IgA in bronchoalveolar lavage (BAL) on PID 7, respectively. BAL samples were titrated, whereas antibody levels in nasal secretions were semi-quantitatively determined and expressed as a percentage of a positive control sample, due to lack of sample material. Standard deviations are indicated by upward deflecting lines. Statistically significant differences between PID 0 and PID 7 in panel A are indicated by a horizontal line, whereas in all panels significant differences between groups for the same time-point are indicated by asterisks and the corresponding group letter; p≤0.05 (*); p≤0.01 (**); p≤0.005 (***); p≤0.001 (****).

Before challenge, on PID 0, BRSV-specific IgA was detected in nasal secretions only from animals immunized with SUMont (Fig. 7A). However, 7 days after BRSV challenge, BRSV-specific IgA was detected in nasal secretions from all vaccinated calves, although, the increase was statistically significant only in those calves that had been vaccinated i.n. with ΔSHrBRSV (Fig. 7A, p<0.01, PID *0 vs. 7*).

Similar to findings in the upper respiratory tract, all vaccinated animals demonstrated significantly higher levels of BRSV-specific IgA in BAL after challenge, compared to controls (ΔSHrBRSV and SUMont p≤0.001; SUAbis p≤0.01; Fig. 7B).

In agreement with the HRSV N-specific serum IgG responses after challenge, animals immunized with either SUMont or ΔSHrBRSV had significantly higher titers of IgA antibodies against HRSV-N in BAL, compared to controls (p≤0.001 and, p≤0.05 respectively; Fig. 7C). Furthermore, titers of HRSV-N specific IgA in BAL from the SUMont calves were also significantly higher than titers in animals in both the ΔSHrBRSV and the SUAbis groups (p≤0.05; Fig. 7C). In contrast to IgA, BRSV specific IgG_1_ antibodies were not detected in BAL or nasal secretions.

#### BRSV-specific cell mediated immune responses

**Figure 8:**
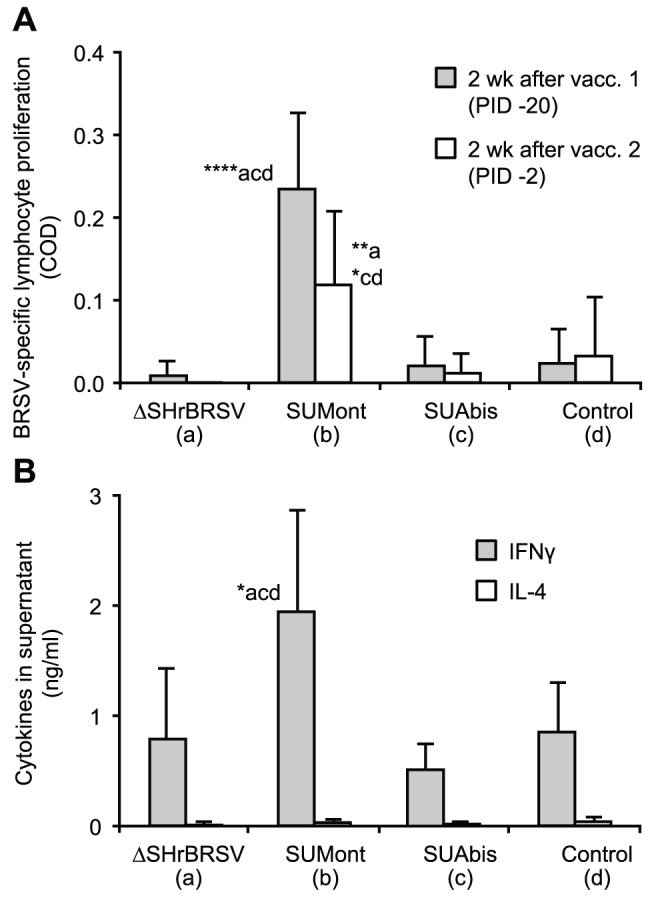
BRSV-specific lymphocyte proliferative response in vaccinated calves. Four groups of 5 calves were vaccinated as described in Fig. 1 and challenged with BRSV, 5 weeks after vaccination, on post-infection day (PID) 0. Two weeks before challenge, one calf (c5) was euthanized due to traumatic injury. Peripheral blood mononuclear cells (PBMC) were purified from blood two weeks after first and second vaccination, as indicated in Fig. 1, and stimulated *ex-vivo* with either BRSV-infected or uninfected cell lysate. (A) Corrected optical density (COD) of Alamar Blue (Invitrogen, Sweden), indicating proliferative response after seven days of incubation. (B) IFNγ and IL-4 in supernatant from PBMC restimulated with BRSV-infected cell lysate, expressed as group means (ng/ml). Standard deviations are indicated by upward deflecting lines. Statistically significant differences are indicated by asterisks and the corresponding group; p≤0.05 (*); p≤0.01 (**); p≤0.001 (****).

BRSV-specific T lymphocyte proliferative responses in PBMCs measured 2 weeks after first and second vaccination, were statistically significant only in animals immunized with SUMont, both after first (p≤0.001) and second vaccination (p≤0.05), compared to all other groups (Fig. 8A). Whereas IL-4 was only detected at very low concentrations (<0.05 ng/ml) in supernatant from restimulated PBMCs from all animals 2 weeks after boost, PBMCs from SUMont-immunized animals produced significant higher levels of IFNγ, compared to those from animals in all other groups (Fig. 8B; p≤0.05; 2 weeks after first vaccination not analysed). Although not detected in PBMCs from ΔSHrBRSV-immunized animals, BRSV-specific proliferative responses were detected in cells from tracheobronchial lymph nodes of these calves, collected 1 week after challenge, and were greater than in cells from controls (Table 3, p≤0.05). These responses were of similar magnitude as those seen in PBMCs from SUMont-immunized animals before challenge (Fig. 8A and Table 3). Lymph nodes from SUMont and SUAbis groups were not analysed, since these could not be prepared following necropsy due to logistical limitations. There was, moreover, a statistically significant increase in proportion of IFNγ-producing CD4^+^ lymphocytes after restimulation of the lymph node cells with BRSV-infected compared to uninfected cell lysate, in ΔSHrBRSV-vaccinated calves (p = 0.02) but not controls (p = 0.14), using paired Student’s *t*-test (Table 3). Despite using inactivated BRSV for restimulation, the proportion of IFNγ-producing CD8^+^ lymphocytes also increased following BRSV-infected compared to uninfected cell lysate stimuli, in ΔSHrBRSV-vaccinated calves (p = 0.06) but not controls (p = 0.4). When IFNγ and IL-4 production was measured in supernatants from BRSV-restimulated LN cells by ELISA, there was no significant difference in IL-4 production, whereas LN cells from ΔSHrBRSV-immunized animals produced significantly more IFNγ, compared to those from controls (p = 0.03; Table 3).

**Table 3:** BRSV-specific lymphocyte responses from tracheobronchial lymph nodes of ΔSHrBRSV vaccinated calves.

	FACS^b^						
	CD4+IFNg+ (%)		CD8+IFNg+ (%)		Lymphocyteproliferation (COD)^c^	ELISA^d^	
Calfgroup^a^	BRSVstim.	Controlstim.	BRSVstim.	Controlstim.		IFNγ (ng/ml)	IL-4 (ng/ml)
ΔSHrBRSV	0.27 (±0.13)*	0.16 (±0.07)*	0.09 (±0.04)	0.06 (±0.02)	0.14 (±0.12)*	1.10 (±1.08)*	0.24 (±0.28)
Control	0.22 (±0.10)	0.19 (±0.13)	0.05 (±0.03)	0.05 (±0.02)	0.02 (±0.03)*	0.20 (±0.10)*	0.32 (±0.27)

a Four groups of 5 calves were vaccinated as described in Fig. 1 and challenged with BRSV, 5 weeks after vaccination, on post-infection day (PID) 0. Lymphocytes were isolated on PID 7 from tracheobronchial lymph nodes of calves vaccinated with ΔSHrBRSV and controls, and stimulated *ex-vivo* with either BRSV-infected or uninfected cell lysate (heat-inactivated).

b After 18 hours of incubation, production of IFN**γ** by CD4+ and CD8+ lymphocytes were assayed using a flow cytometer (FACSVerse, BD Biosciences) and data were analyzed using FACSuite software (BD Biosciences). Results are expressed as % of CD4^+^ or CD8^+^ cells producing IFNγ.

c After 7 days of incubation, proliferative responses were determined by corrected optical density (COD) of Alamar Blue (Invitrogen, Sweden). Results are expressed as the mean corrected OD (COD, OD_BRSV_–OD_cell lysate_).

d After 9 days of incubation, IFNγ and IL-4 were analysed in supernatants of BRSV-restimulated cells by ELISA (BioRad).

*Statistically significant difference between groups is indicated by asterisks; p≤0.05 (*). Standard deviations are presented within parenthesis.

## Discussion

In the present study, two very promising DIVA-compatible BRSV vaccine candidates were identified, when evaluated in young calves with BRSV-specific MDA in a BRSV challenge with severe clinical expression, compared to many published evaluation studies for commercial vaccines [22]. Based on efficacy in reducing clinical signs of disease, consolidated lung lesions and viral load, the vaccine candidates consistently exhibited three distinct levels of protection. ΔSHrBRSV, a live attenuated SH gene-deleted recombinant BRSV, induced almost total clinical protection, and a high level of virological protection, five weeks after a single i.n. immunization. In the same experiment, calves immunized twice i.m. with SUMont, a HRSV subunit vaccine with epitopes from BRSV adjuvanted by Montanide ISA71^VG^, were also well protected, when challenged with virulent BRSV two weeks after the second vaccination. Although high, the protection observed in SUMont immunized animals was not as great as that observed in ΔSHrBRSV immunized animals. In contrast, the same subunits adjuvanted by AbISCO-300 and administered s.c., twice at an interval of three weeks, afforded statistically significant, but limited protection two weeks after the second vaccination.

The strong protection induced by ΔSHrBRSV confirmed previous results obtained in 1 to 4 week-old, BRSV-seronegative calves, vaccinated i.n. and intratracheally with ΔSHrBRSV and challenged with virulent BRSV, 6 months after vaccination [24]. This vaccine virus appears to be attenuated compared to wild type rBRSV by replicating less well in the lower respiratory tract and inducing little or no pathological lesions in 2 to 3 week-old gnotobiotic calves [24]. In the present study, only low levels of virus RNA were detected in nasal swabs from only one out of five conventional calves after vaccination, and furthermore, sentinel calves did not become infected after 6 days of contact with ΔSHrBRSV vaccinated calves. This contrasts with the higher levels of virus shedding detected in the upper respiratory tract in gnotobiotic calves seen in previous studies [24] and might partly be explained by the presence of BRSV-specific MDA, which inhibited virus replication. Altogether, this suggests that the very mild clinical signs observed following i.n. immunization with ΔSHrBRSV were unlikely caused by viral replication, but were probably due to other factors. Even if further studies need to be performed to confirm the good innocuity of ΔSHrBRSV, these observations and the nature of the gene-deletion approach, which makes ΔSHrBRSV more refractory to wild-type reversion compared to live vaccines attenuated by point mutations [23], suggests that the use of ΔSHrBRSV in young calves is safe. The safety of this vaccine, like the adjuvanted SU vaccines, was further confirmed by the absence of exacerbated histopathological lesions or an influx of eosinophils in the lungs, following BRSV challenge of the vaccinated calves, which have been observed with some inactivated vaccines both in the field and experimentally [20], [21].

Surprisingly, BRSV-specific immune responses could not be demonstrated in animals immunized with ΔSHrBRSV, until after challenge with virulent BRSV. Following challenge, the strong protection induced by ΔSHrBRSV was in part associated with rapid and strong anamnestic, local and systemic humoral immune responses. In agreement with previous studies, these were characterized by BRSV-specific IgA in respiratory secretions and BRSV-neutralizing serum antibodies directed against the F and G proteins [5], [10], [12], [13], [18]. Although undetectable in PBMC before challenge, the BRSV-specific T cell responses detected in tracheobronchial lymph nodes after challenge similarly indicated anamnestic cellular responses, which may have contributed to protection. These responses were dominated by IFNγ rather than IL-4 production, partly by CD4^+^ and possibly by CD8^+^ lymphocytes, which are important for BRSV clearance [15].

Whereas ΔSHrBRSV-induced protection seems to have been largely mediated by BRSV-neutralizing systemic antibodies, BRSV-specific local IgA and T cell responses directed against native viral proteins, protection observed in SUMont-vaccinated animals were mediated mainly by T-cell cross-reactions against the internal proteins N, P and M2-1 of HRSV. Unfortunately, we were not able to assess the local T cell immunity in SUMont-vaccinated animals, however, Riffault et al. [25] demonstrated N^SRS^-specific IFNγ production of tracheobronchial lymph node cells in calves vaccinated with N^SRS^ and Montanide ISA71^VG^, compared to unvaccinated controls, 20 days after BRSV challenge. In further agreement with that study, serum antibodies were also induced against these proteins but were not neutralizing, and it is likely that induced mucosal IgA antibodies had similar characteristics. In relation to N^SRS^ evaluated in seronegative calves, protection appears to have been strongly enhanced by the inclusion of additional internal HRSV proteins P and M2-1, with known CD8+ epitopes [14] (G. Taylor, unpublished observations). SUAbis, containing the same subunits but adjuvanted by AbISCO-300, induced immune responses of a similar type but of lower magnitude.

The route of immunization, the presence of BRSV-specific MDA, the composition of the vaccine, and/or the type of adjuvant in the SU vaccines may contribute to the differences in vaccine-induced protection observed in this study. One reason for the superior protection provided by the intranasal administration of a live virus vaccine may be due to the homing-mechanisms of antigen-specific memory lymphocytes that migrate from lymphoid tissue to mucosal effector sites including the site of infection [45]. Immune responses induced by mucosal vaccination are, moreover, considered to be less inhibited by antigen-specific MDA than those induced by parenteral vaccination [5], [13]. One i.n. BRSV vaccine is commercially available for use in young calves with MDA in the field and seems effective in this target animal group, but it is not DIVA compatible. Despite the limited virus replication following i.n. vaccination with ΔSHrBRSV, and the absence of a detectable BRSV-specific immune response before challenge in this study, BRSV-specific MDA did not appear to inhibit priming of protective immunity induced by ΔSHrBRSV.

The superior protection induced by ΔSHrBRSV may be explained by the expression of full-length glycoproteins with the native conformation, even if they were expressed in low quantities due to limited viral replication. In addition, the virus replication and presence of viral pathogen-associated molecular patterns would be expected to activate the innate immune system and to present antigens on major histocompatibility complex class I, which will initiate a CTL response.

The antigenic epitopes used in the SU vaccines in the present study, were carefully selected epitopes from the F and G proteins, which were grafted onto N nanorings. Specifically, F_422–438_ corresponds to a linear epitope on the fusion protein, antigenic site IV, which is the target of MAb19 [38] and 101F [46], and is also recognized by a protective, BRSV-neutralizing bovine mAb [47] (P Whyte & G Taylor, unpublished observations). MAb19 also binds with high affinity to F_mimo_, a combinatorial peptide mimicking the same epitope, and which was reported to induce neutralizing antibodies [37]. F_255–278_ corresponds to antigenic site II on F, which is the target of Palivizumab and Motavizumab, and is recognized by a protective BRSV-neutralizing mAb [38], [47]. G_174–187_ corresponds to a dominant protective epitope on the attachment protein, which induces partially protective, but non-neutralizing, antibodies in calves [48]. These epitopes have not been shown to be T-cell epitopes in cattle [49], but their contribution to cellular immunity is possible and needs to be further elucidated. All purified nanorings with grafted epitopes were recognized by the respective epitope-specific monoclonal antibody, as determined by ELISA (data not shown). However, antibodies detected by ELISA in animals immunized with SUMont were directed against HRSV N, P and M2-1 but not against F and G. The lack of antibodies directed against the F and G proteins in calves immunized with SU might be explained by problems of conformation or accessibility, or by the relatively low quantity of these epitopes in the SU preparations, compared to the quantity of full-length HRSV-N, -P and -M2-1. Indeed, a recent study demonstrated enhanced immunogenicity, when the influenza epitope (M2e) attached to N-nanorings were repeated [50].

Increases of N-, P- and M2-1-specific IgG in the SU-vaccinated animals were detected after vaccination but were not evident by measuring total BRSV-specific serum IgG_1_ antibodies. This might be explained by a masking effect of declining MDA specific to other proteins (*e.g.* BRSV F [8]) after first vaccination, or differences in test sensitivity, possibly due to differences in the amount of these proteins in ELISAs based on BRSV-infected lysate and recombinant proteins, respectively. The contribution of N-, P- and M2-1-specific antibodies to SU-induced protection should be marginal, since these are all internal virus proteins. Taken together, these findings suggest that the F and G epitopes played a very limited role in the protection observed in SU-vaccinated calves and that cross-protective T-cell responses are induced by HRSV-N in calves, as previously described [25] and likely also by P and M2-1. Indeed, N, P and M2-1 are highly conserved, with 93%, 81% and 80% amino acid homology between BRSV and HRSV, respectively [51]–[53], which strengthens the possibility that all of them could have contributed to the observed cross-protection. This level of cross protection has not been observed in previous investigations with live RSV in different animal species. Immunization with BRSV provided cotton rats with limited protection against HRSV challenge [54] and recombinant BRSV with glycoproteins F and G from HRSV was overly attenuated in chimpanzees, with marginal viral replication, humoral response and protective efficacy [55]. Likewise, HRSV replicates poorly in calves and induces only mild lung lesions after intranasal and intratracheal administration [56] (Valarcher & Taylor, unpublished observations). Therefore the lack of viral replication due to the species barrier, which might explain a poor protective immune response, can be bypassed by direct administration of conserved recombinant viral proteins combined with a powerful adjuvant, as demonstrated by SUMont in the present study.

The SUMont-immunized animals were the only calves that demonstrated a systemic BRSV-specific proliferative T-cell response after first and second vaccination, with production of IFNγ. The F, N, P and M2 proteins are the major antigens recognized by CD8+ T cells [14] (Taylor unpublished observations) and N, P and M2-1 were present in high quantities in the SU vaccines. Recall responses in PBMCs stimulated with either BRSV lysate or N^SRS^ have been observed in calves immunized with N or N^SRS^ alone [10], [25]. In the present study, however, only BRSV lysate restimulation of PBMCs was performed, so the contribution of the individual proteins to T-cell priming was not determined. Nonetheless, our data suggest that the T-cell responses plays a very important role in the protection against RSV and that SUMont could be a good base for the development of a vaccine against BRSV as well as HRSV. Further improvement of the protective efficacy could be likely obtained by including the pre-fusion F-protein of BRSV or HRSV [57] instead of decorating N with epitopes from F and G.

Not only the proteins included in SU but also the adjuvant played an important role in the efficacy of these vaccines. The adjuvant effects of water-in-oil emulsion vaccines, such as SUMont, are not fully understood, but include the induction of inflammation and recruitment of cells to the site of immunization, as well as a depot effect [58]. In this work, IFNγ was detected in the supernatant from BRSV-stimulated PBMC collected from animals vaccinated with SUMont after boost, but only minimal amounts of IL-4, suggesting a T helper cell type 1 (Th 1) orientation of the immune response. Although not confirmed herein, Iscomatrices such as AbISCO-300, is similarly known to induce Th1 responses and prime CTL by antigen cross-presentation on dendritic cells and B-cells [59], [60] and activate dendritic cells through recruitment of IFNγ-producing NK cells to draining lymph nodes [61]. The limited protection induced by SUAbis in the present study, contrasts with the high protective efficacy of s.c. administered classic BRSV-ISCOMs, containing similar adjuvant quantities [12], [62], but additionally the F, G, SH and M proteins [63].

The difference in protection between SUMont and SUAbis may be explained by the use of two different route of immunization. However, to our knowledge, the subcutaneous route of immunization has not previously shown to be disadvantageous in cattle.

Combining different types of vaccines, adjuvants and routes of administration, in a heterologous prime-boost, may be a way to improve the efficacy as well as the duration of protection induced by vaccination. Although not evaluated herein, the duration of protection might be limited after a single mucosal administration [64], and theoretically, a homologous mucosal boost of ΔSHrBRSV might be ineffective, due to vaccine neutralization by secretory IgA antibodies. Boosting intramuscularly with ΔSHrBRSV or SUMont could thus prolong and potentiate protective immunity, by activating several arms of the immune system.

Finally, one characteristic of this combined or separate vaccine approach is to enable DIVA, by measuring antibodies against a protein that is absent in the vaccine. The concept was introduced as a way to implement disease eradication programs in veterinary medicine, to limit the spread of a disease, while not being serologically blinded by vaccination [65]. However, the DIVA-aspect can also be used at a population level in the field to serologically monitor the virological protection induced by vaccination, in cattle as well as in man, since vaccine efficacy and duration of protection may change over time due to genetic evolution of field strains [66]. The envisaged DIVA-target in the present study is the SH protein, which has been excluded from both vaccines: by genetic manipulation in ΔSHrBRSV, and by rational design in the subunit vaccines. As serum antibodies against SH are induced by natural BRSV infection [63], detection of these antibodies will presumably enable the differentiation of infected animals from those vaccinated with either vaccine in the present study. In calves, the detection of IgG_2_ antibodies would increase the specificity, since this isotype is not present in high quantities in MDA [13].

In conclusion, our data suggest that several types of immune response, influenced by vaccine composition and vaccination regimen, may provide protection against BRSV in calves with MDA. A single intranasal administration of ΔSHrBRSV was sufficient to safely induce anamnestic neutralizing systemic and mucosal IgA responses as well as local T cell immune responses affording almost complete protection against BRSV challenge, 5 weeks later. In contrast, SUMont induced good protection in absence of neutralizing antibodies, possibly through strong cross-reactive T-cell responses against the recombinant HRSV proteins N, P and M2-1. We believe that the combination of both vaccines (live and inactivated) in a heterologous prime-boost regimen might afford sterilizing long lasting protection against BRSV and this hypothesis is under evaluation.
